# *In silico* and *in vitro* inhibition of host-based viral entry targets and cytokine storm in COVID-19 by ginsenoside compound K

**DOI:** 10.1016/j.heliyon.2023.e19341

**Published:** 2023-08-20

**Authors:** Vinothini Boopathi, Jinnatun Nahar, Mohanapriya Murugesan, Sathiyamoorthy Subramaniyam, Byoung Man Kong, Sung-Keun Choi, Chang-Soon Lee, Li Ling, Dong Uk Yang, Deok Chun Yang, Ramya Mathiyalagan, Se Chan Kang

**Affiliations:** a Graduate School of Biotechnology, College of Life Sciences, Kyung Hee University, Yongin-si, Gyeonggi-do 17104, South Korea; b Department of Oriental Medicinal Biotechnology, College of Life Science, Kyung Hee University, Yongin-si, Gyeonggi-do 17104, South Korea; c Daedong Korea Ginseng Co., Ltd, 86, Gunbuk-ro, Gunbuk-myeon, Geumsan-gun, Chungcheongnam-do 32718 Republic of Korea; d Research and Development Center, Insilicogen Inc., Yongin, Republic of Korea

**Keywords:** Ginsenoside, Compound K, ACE-2, TMPRSS2, Molecular docking, Molecular dynamics simulation, Raw 264.7, CaCo-2, A549, MCF7

## Abstract

SARS-CoV-2 is a novel coronavirus that emerged as an epidemic, causing a respiratory disease with multiple severe symptoms and deadly consequences. ACE-2 and TMPRSS2 play crucial and synergistic roles in the membrane fusion and viral entry of SARS-CoV-2 (COVID-19). The spike (S) protein of SARS-CoV-2 binds to the ACE-2 receptor for viral entry, while TMPRSS2 proteolytically cleaves the S protein into S1 and S2 subunits, promoting membrane fusion. Therefore, ACE-2 and TMPRSS2 are potential drug targets for treating COVID-19, and their inhibition is a promising strategy for treatment and prevention. This study proposes that ginsenoside compound K (G-CK), a triterpenoid saponin abundant in *Panax Ginseng*, a dietary and medicinal herb highly consumed in Korea and China, effectively binds to and inhibits ACE-2 and TMPRSS2 expression. We initially conducted an *in-silico* evaluation where G-CK showed a high affinity for the binding sites of the two target proteins of SARS-CoV-2. Additionally, we evaluated the stability of G-CK using molecular dynamics (MD) simulations for 100 ns, followed by MM-PBSA calculations. The MD simulations and free energy calculations revealed that G-CK has stable and favorable energies, leading to strong binding with the targets. Furthermore, G-CK suppressed ACE2 and TMPRSS2 mRNA expression in A549, Caco-2, and MCF7 cells at a concentration of 12.5 μg/mL and in LPS-induced RAW 264.7 cells at a concentration of 6.5 μg/mL, without significant cytotoxicity.ACE2 and TMPRSS2 expression were significantly lower in A549 and RAW 264.7 cells following G-CK treatment. These findings suggest that G-CK may evolve as a promising therapeutic against COVID-19.

## Graphical abstract

**Figure:**
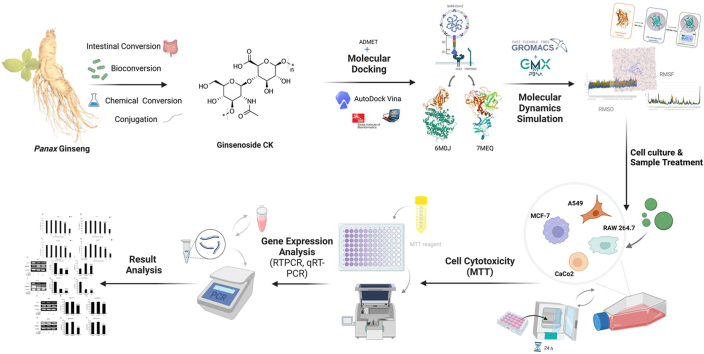

## 1 Introduction

In December 2019, the World Health Organization declared a zoonotic virus called SARS-CoV-2, later named Covid-19, which originated in Wuhan, China. By March 2020, it was declared a pandemic emergency [1]. Since then, Covid-19 has rapidly spread to over 200 countries. Compared to other coronaviruses, the genome of SARS-CoV-2 is 79.5% identical to SARS-CoV-1 and 96.2% similar to a bat coronavirus [2]. SARS-CoV-2 is a non-segmented positive-sense, single-stranded ribonucleic acid (RNA) virus consisting of approximately 30 kb nucleotides. It has four structural proteins: nucleocapsid (N), membrane (M), envelope (E), and spike (S) [3]. During SARS-CoV-2 infection in humans, the initial crucial step is the interaction between the viral S protein and cellular host receptors. The S protein consists of two functional domains, S1 and S2, similar to other coronaviruses [4]. The S1 subunit, which contains the receptor-binding domain (RBD), plays a vital role in identifying and binding target receptors. It consists of N-terminal and C-terminal domains. On the other hand, the S2 subunit, with a transmembrane domain, is responsible for the membrane fusion process [5]. Among the various receptors targeted by the virus, the primary target of the S1 subunit's RBD is the Angiotensin-converting enzyme 2 (ACE2) receptor.

ACE2, a vital element of the renin-angiotensin system (RAS), is primarily found in epithelial cells of various organs, including the small intestine, heart, testis, kidney, trachea, alveoli, bronchi, respiratory tract monocytes, and alveolar macrophages. The RAS comprises two arms: one that produces angiotensin II (Ang II) from angiotensinogen and another that breaks down Ang II into angiotensin 1-7 (Ang 1–7) through ACE2. ACE2 is a counter-regulatory mechanism inhibiting the RAS system by eliminating Ang II. ACE2 expression is elevated in specific individuals due to various factors. In comorbidities associated with severe and high-risk COVID-19 cases, such as COPD (Chronic obstructive pulmonary disease), smoking, air pollution [6], cardiovascular diseases (CVD), and type 2 diabetes (T2D), ACE2 levels are increased in the lungs, heart, and oral cavity. Consequently, aging individuals and those with cardiovascular and metabolic conditions experience more severe respiratory distress, myocardial injury, renal failure, and increased mortality from SARS-CoV-2 infection [7,8]. ACE2 plays a crucial role in the pathogenesis of severe acute lung injury, as downregulating its expression reduces lung damage, regulates the renin-angiotensin pathway, and mitigates acute respiratory distress syndrome (ARDS) symptoms. The use of ACE inhibitors (ACEIs) or angiotensin receptor blockers (ARBs) in hypertension treatment has been linked to increased ACE2 expression [[9], [10], [11]]. However, the impact of ACEIs and ARBs on COVID-19 infection is still under debate, as they may have both facilitating (viral entry) and protective effects. Therefore, a holistic approach is necessary when targeting ACE2 as a therapy for COVID-19, considering the infection stage, inflammation, coagulation status, and disease progression for each patient. Strategies to reduce viral infectivity by suppressing the ACE2 expression are prioritized during the initial infection stage while enhancing ACE2 activity becomes appealing in advanced stages with exuberant inflammation. While ACE and ACE2 share amino acid homology, their active sites differ, and ACE inhibitors do not directly affect ACE2 activity but can activate it. Currently, no drugs are available to modulate ACE2 expression directly, although NAAE is an ACE2 inhibitor that inhibits SARS-CoV spike protein-mediated cell fusion but also inhibits ACE2 catalytic activity. An alternative strategy is using soluble ACE2 (sACE2) as a decoy receptor to trap the virus, but technical challenges need to be addressed, including reducing immunogenicity and maintaining stability [12]. Various studies have explored the inhibitory potential of small molecules on ACE2, considering stability concerns for protein or peptide drugs.

A multifaceted approach is vital when evaluating the potential of drugs, as focusing solely on ACE2 may not be sufficient to prevent severe COVID-19 outcomes. Another important target in combating SARS-CoV-2 is TMPRSS2, which contributes to viral infection and cell entry alongside the ACE2 receptor. TMPRSS2 is highly expressed in multiple organs and plays a significant role in the detachment of functional domains during the virus's entry into cells. Therefore, Transmembrane serine protease 2 (TMPRSS2) is an essential target in combating SARS-CoV-2 as it plays a significant role in viral infection and cell entry [13]. TMPRSS2, known for activating influenza and SARS-CoV, also contributes to the infection process of SARS-CoV-2 in addition to the ACE2 receptor [14]. Structurally, TMPRSS2 protease is composed of 492 amino acids and includes a 70-amino acid N-terminal region, a 36-amino acid transmembrane domain, an LDL receptor class A domain (LDLRA), a scavenger receptor cysteine-rich (SRCR) domain, and a serine protease domain connected to an activation domain through a disulfide bond. TMPRSS2 exhibits high expression in various organs, such as the breast, kidney, pancreas, bile duct, prostate epithelial cells, stomach, ovary, small intestine, lung, salivary glands, and colon [15]. After the virus binds to the ACE2 receptor, proteolytic cleavage typically occurs at the S1/S2 boundary or within the S2 domain. This detachment of functional domains occurs in the plasma membrane, facilitating rapid virus entry into cells through a pH-independent mechanism [16,17]. Therefore, inhibiting the expression of TMPRRS2 will be crucial. In addition, factors like immune response, inflammation, and comorbidities also play significant roles and should be considered. On the other hand, the role of ACE2 and TMPRSS2 in cancer during SARS-CoV-2 infection should be explored to understand the severe infectivity and high mortality rates in respective patients suffering from various types of cancer.

Viral-based experiments have limitations in capturing the complexity of human physiology, while host-based experiments using human samples offer a more accurate representation. However, viral-based experiments may only partially capture the range of crucial host immune responses necessary for understanding COVID-19 outcomes. They also limit the generalizability of findings by focusing on specific strains or variants, whereas host-based experiments capture the natural diversity of the virus. Safety concerns arise from working with live SARS-CoV-2 in viral-based experiments. In contrast, host-based experiments involve fewer risks, are clinically relevant, and significantly impact therapeutics, vaccines, disease severity factors, and public health strategies. A combination of approaches is necessary to gain a comprehensive understanding of COVID-19, integrating viral-based and host-based methods. Therefore, this study focuses on host-based experiments. Due to the rapid spread of SARS-CoV-2, researchers are repurposing FDA-approved drugs and utilizing computational biology techniques to identify new treatments [[18], [19], [20], [21], [22]]. *In silico* studies, followed by *in vitro* and *in vivo* validations, streamline the drug discovery process [[23], [24], [25], [26], [27]]. Additionally, exploring naturally available medicinal plants offers a promising source of therapeutic drugs. This integrated approach addresses the urgent need for effective and cost-efficient treatments for SARS-CoV-2 [[28], [29], [30]]. Several natural compounds have recently been screened for their inhibitory potential against COVID-19 targets [[31], [32], [33]].

Among the naturally available medicinal plants, ginseng, belonging to the Araliaceae family, particularly *Panax ginseng*, has gained attention for its therapeutic potential [34,35]. Ginseng contains bioactive components such as ginsenosides, polysaccharides, phytosterols, and glycosides, which possess pharmacological properties [36]. More than 150 identified ginsenosides significantly treat various conditions, including cancer, inflammation, cardiovascular diseases, autoimmune disorders, and metabolic conditions [[37], [38], [39]]. Recent studies have explored the antiviral efficacy of fermented black ginseng extract in inhibiting SARS-CoV-2 replication and reducing viral RNA copies [40]. Other studies have also investigated the antiviral potential of ginseng against SARS-CoV-2 [[41], [42], [43], [44], [45], [46]]. A study in 2018 demonstrated the glucocorticoid-like activity of Protopanaxadiol (PPD) and Protopanaxatriol (PPT), which showed potent and selective GR agonist properties [47]. The ability of ginsenosides to regulate the non-genomic effects of GR has also been examined [48]. The extensive utilization and efficacy of steroid receptor ligands in various lung diseases have prompted trials in COVID-19 patients experiencing severe symptoms [49]. Remarkably, dexamethasone has reduced mortality among COVID-19 patients requiring respiratory support, leading to its widespread adoption as a standard treatment for hospitalized COVID-19 patients worldwide. Its primary mechanism of action seems to involve inhibiting the production of cytokines, thereby mitigating the cytokine storm. This treatment has demonstrated effectiveness in reducing viral loads and promoting patient recovery, although there are potential risks associated with concurrent immunosuppression. Studies on mouse placentas have indicated that dexamethasone inhibits ACE2 expression. Additionally, dexamethasone has been observed to inhibit TMRPSS2 expression in the A549 lung cell line. It is plausible that steroid receptors, aside from modulating the immune system, might also influence the expression of ACE2 and TMPRSS2, potentially impacting the uptake and infection of SARS-CoV-2. Dexamethasone, for instance, inhibits the entrance of the SARS-CoV-2 spike pseudo-typed virus into the cell by binding to ACE2 [50]. The multifaceted impact of dexamethasone suggests a potential interplay between steroid receptors and viral entry proteins in COVID-19 infection, although this relationship is currently unknown. Nevertheless, emerging preclinical data suggest that targeting both receptors simultaneously could be advantageous [51]. Despite the promise of drugs like dexamethasone and remdesivir in treating severe COVID-19 cases, their side effects necessitate the exploration of alternative natural inhibitors targeting viral proteins [52,53].

**Fig. 1:**
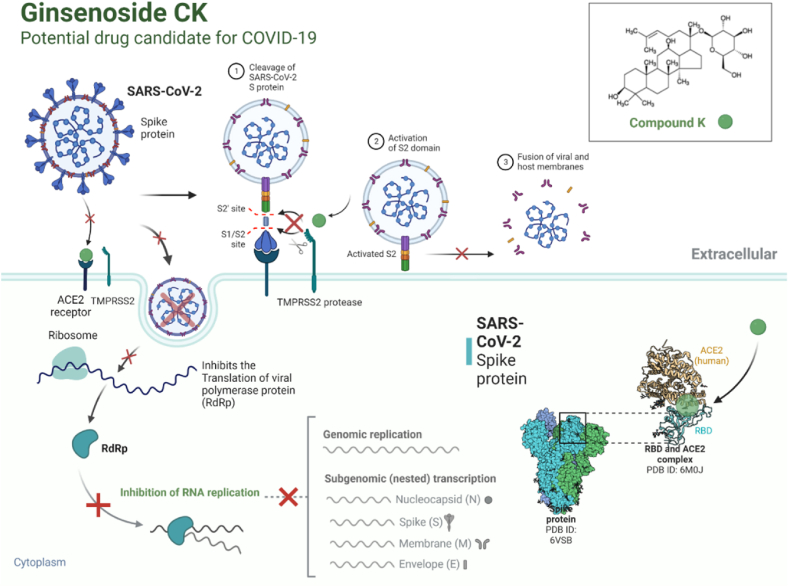
Illustration of the mechanism of action of G-CK against the main drug targets (ACE2 and TMPRSS2) of COVID-19.

Thus, naturally occurring small molecules that mimic the action of dexamethasone hold promise. Ginsenosides have been studied for their anti-inflammatory properties and have shown potential in modulating immune responses. Their structural similarity to dexamethasone, a synthetic corticosteroid, suggests that they have similar mechanisms of action. However, the potential of G-CK, a naturally occurring from *Panax ginseng,* in modulating the expression of COVID-19 related targets remains unexplored. G-CK, a primary metabolite derived from the oral administration of PPD-type ginsenosides, exhibits higher biological activity and has been the focus of conversion studies to enhance the potency of ginsenosides [[54], [55], [56]]. This study aims to investigate the inhibitory effects of G-CK derived from *Panax ginseng* on ACE2 and TMPRSS2 proteins of SARS-CoV-2 using molecular docking techniques. Additionally, molecular dynamics (MD) simulations and free energy calculations were conducted to assess the stability of G-CK. In vitro cytotoxicity and gene expression analyses were performed on A549, Caco-2, and MCF7 cell lines to validate the findings obtained through computational modeling. Furthermore, the study investigated the potential inhibitory effects of G-CK on ACE2, TMPRSS2, and cytokine (*COX-2, TNF-α, iNOS, IL-6, and IL-8*) expressions induced by LPS. Dexamethasone, a drug with multiple roles in managing COVID-19 and other diseases, was employed as a control in this study. The results indicate that G-CK shows promise as a potential effective treatment for COVID-19, as illustrated in Fig. 1.

## 2 Materials and methods

### 2.1 Ligand and protein preparation

For the Present study, we have selected G-CK, a triterpenoid saponin belonging to *P.ginseng*. The 3D chemical structure of G-CK was retrieved from PubChem [57]. The ligand molecules were further processed and converted to the required pdbqt format using Autodock tools [58]. The drug targets of SARS CoV-2 were identified based on the literature survey, database search, and knowledge of their role in pathogenicity, especially in mediating host cells. The significant targets involved in the host entry virulence mechanism of SARS-CoV-2 are ACE2 (PDB ID:6MOJ) [59] and TMPRSS2 (PDB ID:7MEQ). The selected proteins' three-dimensional (3D) structures are available in their native forms and were downloaded from the RCSB PDB database [60] in the PDB format. The structure preparation consists of several steps, such as deleting all water molecules and inhibitors, checking and repairing the missing atoms, and adding hydrogens and required charges using Autodock tools. The co-crystallized ligand such as N-Acetyl-d-Glucosamine (NAG) and nafamostat from the native PDB structures of the target protein was used as a reference standard along with a few other selected control drugs such as dexamethasone, remdesivir, camostat, indinavir, and chloroquine.

### 2.2 Active site identification

Although the binding site is well characterized for ACE2 and TMPRSS2 as they are crucial drug targets for the new SARS-CoV-2. We have applied the DoGSiteScorer online tool to compare and crossverify the predicted binding pockets within native ACE2 and TMPRSS2 with the already reported binding sites. DoGSiteScorer is a grid-based method that uses a Difference of Gaussian filter to detect potential binding pockets solely based on the 3D structure of the protein and splits them into subpackets. Global properties are calculated, describing the size, shape, and chemical features of the predicted (sub) pockets. Per default, a simple draggability score is provided for each (sub) pocket based on a linear combination of the three descriptors describing volume, surface, hydrophobicity, and enclosure. The binding pockets are ranked according to size, surface area, and draggability score [61].

### 2.3 Molecular docking studies

Autodock Vina provides better ligand-protein binding poses and accuracy than AutoDock 4.2. Hence, we have used AutoDock Vina [62] for this study's docking experiments to dock the selected ligand molecule G-CK and seven control drugs against ACE2 and TMPRSS2. The Supplementary File (S2) contains the gird size and exhaustiveness number information for all the docking procedures. All Compounds were further docked to obtain docking scores and the binding sites. Finally, we have utilized the academic version of PyMOL [63] MAESTRO 13.3 (Schrödinger, LLC, NY, USA), and BIOVIA Discovery Studio Visualizer (BIOVIA, Dassault Systèmes) [64] to visualize the ligand interactions with the active sites of the receptors.

### 2.4 Molecular dynamics (MD) simulation studies

The stability of the ACE2-TMPRSS2 with G-CK complex was assessed by performing Molecular Dynamics Simulation (MDS). The MDS for the G-CK and Dexamethasone with ACE2 and TMPRSS2 was performed for 100 ns using GROMACS 2020.1 software [65]. The ACE2 and TMPRSS2 protein topology files were created using the gromacs utility ((pdb2gmx)). We utilized Swiss PARAM to produce the topologies of drugs [66]. The receptor-ligand complexes were placed in a periodic cubic solvated box, and an explicit SPC water model solvated the cell. Appropriate counter ions such as Na or Cl were used to neutralize the system. Energy minimization and equilibration for all systems were executed using NVT and NPT ensembles. Further, 50 nano-second (ns) production was carried out individually for all systems. Finally, we performed the MD simulation for 100 ns time steps. Next, we used the output data such as RMSD, RMSF, Rg, and the number of hydrogen bonds to analyze MD trajectories using the GROMACS utilities. The root mean square deviation (RMSD) of certain atoms in a molecule with respect to a reference structure can be calculated with the program gmx rms by least-square fitting the structure to the reference structure (t2 = 0) and subsequently calculating the RMSD. The RMSD values are calculated using the following formula (1).

(1) $$RMSD\left(t_{1,}t_{2}\right)={\left[\frac{1}{M}\sum_{i=1}^{N}{m_{i}\left\|r_{i}\left(t_{i}\right)-r_{i}\left(t_{2}\right)\right\|}^{2}\right]}^{\frac{1}{2}}$$

Where $M=\sum_{i=1}^{N}m_{i}and\;r_{j}\left(t\right)$ is the position of atom I at time t. Note that fitting does not have to use the same atoms as the calculation of the RMSD; e.g. a protein is usually fitted on the backbone atoms (N,C:math:_{alpha},C), but the RMSD can be computed of the backbone or of the whole protein.

The Root Mean Square Fluctuation (RMSF) is a significant aspect of Molecular Dynamics (MD) that can be studied. It enables the analysis of the fluctuations exhibited by an individual atom or a group of atoms, such as a protein residue, throughout a simulation. By assessing the RMSF, we can identify the regions within our system that display the greatest mobility. This information provides valuable insights into the structural dynamics of the system. To calculate the RMSF, we determine the square root of the variance in the fluctuations around the average position. Essentially, we measure the root mean square of the deviations in atomic positions from their mean positions across a series of time steps in a molecular dynamics’ simulation. For a specific atom *I,* we can express this as follows (2):

(2) $${RMSD}_{i}=\sqrt{\left\langle {\left(r_{i}-\left\langle r_{i}\right\rangle \right)}^{2}\right\rangle }$$

In the equation, r_i_ represents the coordinates of atom *i,* while < *r*_*i*_> represents its average position within the ensemble. Interpreting the RMSF value is relatively simple. Regions with high RMSF values indicate a significant deviation from the average position, suggesting a high level of structural mobility in those areas. Conversely, regions with low RMSF values exhibit minimal deviation from the average position, indicating a greater rigidity during the simulation.

### 2.5 MM_PBSA free binding energy calculation

Finally, we have employed the gmx_MMPBSA [67] package for free energy calculations based on the single trajectory of GROMACS with an appropriate force field. This tool allows free energy calculations using MM/PBSA or GBSA (Molecular Mechanics/Poisson-Boltzmann or Generalized Born Surface Area) methods with an implicit solvent model. The equation provided was utilized to determine the value of ΔGbinding, which represents the binding energy of the protein complexes associated with G-CK.

ΔGbinding=Gcomplex−(Gprotein+Gligand)

This calculation involved subtracting the sum of the protein energy (Gprotein) and ligand energy (Gligand) from the energy of the lead phytochemical/standard inhibitor bound test protein complex (Gcomplex). Gcomplex refers to the energy of the protein complex, while Gprotein and Gligand represent the energy of the protein and ligand in an aqueous environment, respectively.

### 2.6 ADMET analysis

The determination of ADMET characteristics is critical for rolling out unfavorable effects of a drug candidate at the early stages of the drug development process. Therefore, we have employed the ADMETlab 2.0 [68] web server to predict the ADME, physicochemical properties, and toxicity properties of the compounds used in this study. SMILES strings of the selected ligands were submitted to the ADMETlab 2.0 server, and it returned ADMET attributes in pdf and spreadsheet format, which could be downloaded for further analysis.

### 2.7 Chemical reagents for *invitro* studies

Three different cell lines were used in this experimental study, namely the human lung cancer (A549) cell line, breast cancer (MCF 7) cell line, human colorectal adenocarcinoma (Caco-2) cell line, and normal murine macrophage RAW 264.7 cells. All the cell lines were purchased from the Korean cell line bank (KCLB, South Korea). The Caco-2 cell line was grown in the Minimum Essential Medium (MEM), whereas the other cell lines were grown in Roswell Park Memorial Institute (RPMI) 1640 medium. On the other hand, Raw 264.7 cells were grown in Dulbecco's Modified Eagle's Medium (DMEM) media. In addition, the fetal bovine serum and penicillin-streptomycin are added to the media for supplements and antibiotics. The media and the serum were obtained from Welgene Inc., Gyeongsan-si, South Korea.

### 2.8 In-vitro cytotoxicity

Human lung cancer (A549) and breast cancer (MCF 7) cell lines were cultured in round Petri dishes separately using the same amount of FBS and penicillin-streptomycin as before [69,70]. In addition, normal RAW 264.7 cells and human colorectal adenocarcinoma (Caco-2) cell line was grown in a 75-T flask with the same volume of serum and antibiotics. Each cell line was trypsinized and seeded to the 96 well plates at 1 × 10^4^ cells/well density and allowed to grow in a humidified incubator at 37 °C with 5% CO2. Once the 80% confluency was reached, each cell line was treated separately with G-CK with different concentrations (0,3.125,6.25,12.5,25) μg/mL and 10 μM of dexamethasone as a positive control. After 24 h, 20 μl of MTT solution (5 mg/mL, in PBS; Life Technologies, Eugene, OR, USA) were added to the treated cells and the control; incubated for 3 h. The MTT reagent entered the viable cells and turned into purple-colored crystal formazan. To dissolve the crystal formazan, DMSO was added to the cells, and the viability of the cells was obtained using the Enzyme-Linked Immunosorbent Assay (ELISA) reader at 570 nm (Bio-Tek, Instruments, Inc., Winooski, VT, USA).

### 2.9 Effect of G-CK on gene expression of ACE2 and TMPRSS2

Total RNA from RAW 264.7, A549, MCF-7, and Caco-2 cells was obtained using QIAzol lysis reagent (QIAGEN, Germantown, MD, USA). The reverse transcription process was carried out using 1 μg of RNA in 20 μL of amfiRivert reverse transcription reagents (GenDepot, Barker, TX, USA), as directed by the manufacturer. The obtained cDNA was amplified with the following primers *ACE2*, forward: 5′- TCC GTC TGA ATG ACA ACA GC -3′ and reverse: 5′- CAC TCC CAT CAC AAC TCC AA -3′, *TMPRSS2*, forward: 5′- GAG GAC GAG AAT CGG TGT GT -3′ and reverse: 5′- TCC AGT CGT CTT GGC ACA -3′, *COX-2*, forward: CCT GAG CAT CTA CGG TTT GC and reverse: ACT GCT CAT CAC CCC ATT CA, iNOS, forward: CCT GAG CAT CTA CGG TTT GC, and reverse: GGCCTAAGGTCCACTTGTGTCA, *TNF-α*, forward: GCCAGAATGCTGCAGGACTT and reverse: GGCCTAAGGTCCACTTGTGTCA, IL-6, forward: AGGGTTGCCAGATGCAATAC and reverse: AAACCAAGGCACAGTGGAAC, IL-8, forward: CCGGAGAGGAGACTTCACAG and reverse: GGAAATTGGGGTAGGAAGGA, GAPDH, forward 5′‐CAA GGT CAT CCA TGA CAA CTT TG‐3′ and reverse 5′‐GTC CAC CAC CCT GTT GCT GTA G‐3′. The reaction was repeated 3 times for 30 s at 95 °C, 30 s at 60 °C, and 50 s at 72 °C. The amplified RT-PCR results were examined on 1% agarose gels, stained with Safe Pinky DNA Gel Staining (GenDepot, Barker, TX, USA), and captured under UV light.

## 3 Result and discussion

### 3.1 Active site identification of ACE2 and TMPRSS2

**Fig. 2:**
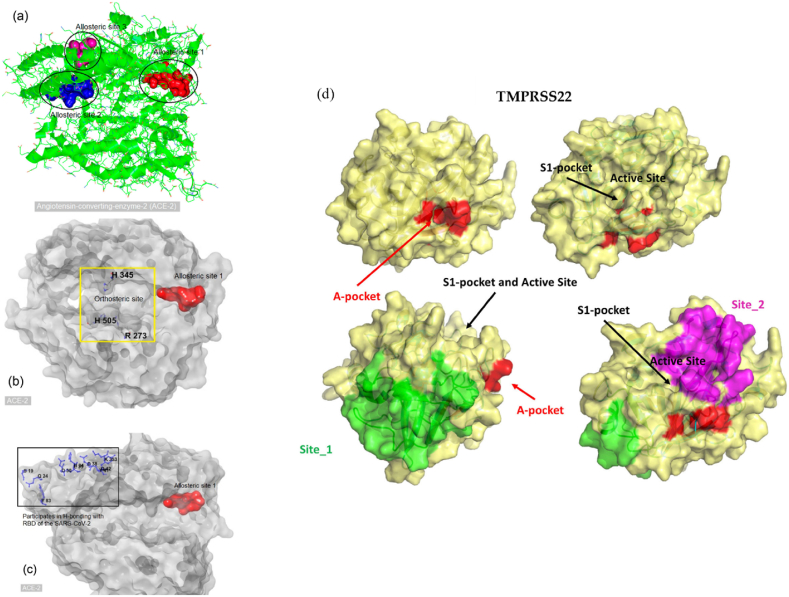
The allosteric sites of angiotensin-converting enzyme 2. (a) Cartoon presentation of the human angiotensin-converting enzyme 2(3SCJ). Different black shapes highlight potential allosteric areas. (b) A yellow square highlights the orthosteric site of ACE-2. Essential amino acid residues are H345, H505, and R273. (c) Allosteric site 1 (red) and amino acid residues (blue) of ACE-2 participating in hydrogen bonding (H-bond) with the receptor-binding domain (RBD) of SARS-CoV-2 (d) Visual summary of all the possible binding sites of TMPRSS2. Adapted from Refs. [71,73].

Generally, two types of drug targets are available when an enzyme plays a critical role in a particular disease. The two types are an orthosteric site and an allosteric site of an enzyme. Drugs designed for the orthosteric site usually bind to the enzyme's active site via competitive, uncompetitive, or noncompetitive inhibitions. Drugs for allosteric sites bind to sites other than the active site of the enzyme and often alter the shape of the active site; that is, they can allosterically alter the conformation of the protein. Allosteric drugs can alter the biophysical properties of an enzyme. In addition, some allosteric drugs act as molecular switches, whereby a slight structural change (altered biophysical properties) disturbs the mechanism of protein−protein interactions. Similarly, alteration of the biophysical properties of the ACE-2 and TMPRSS2 receptor could be possible upon binding of an allosteric drug, which may disrupt the interactions between ACE-2, TMPRSS2, and the RBD of SARS-CoV-2. In particular, binding a drug at the allosteric site of the ACE-2 and TMPRSS2 receptor may decrease biophysical interactions (e.g., electrostatic, hydrogen bonding) with the viral RBD. Hence, altering the biophysical properties of the ACE-2 and TMPRSS2 receptors by modulating an allosteric site of ACE-2 and TMPRSS2 may be a promising strategy against COVID-19 [71]. Firstly, the allosteric sites for both ACE2 [59,71,72] and TMPRSS2 [73,74] have been taken from the previously reported literature and represented in Fig. 2(a–d) and Table S2. Secondly, we have applied the DoGSiteScorer online tool to predict binding pockets within native ACE2 and TMPRSS2, and the results have been represented in Table S3 and Fig. S1. These active sites and binding pocket identification are vital in validating the above-mentioned experimentally reported active sites and the results from the current study, which will be beneficial in confirming the inhibitory potential of G-CK against SARS-CoV-2. Interestingly, the active sites from the literature studies and the predicted sites from the DoGSiteScorer online tool were almost similar, confirming the accuracy of the prediction tool.

### 3.2 Molecular docking analysis

#### 3.2.1 Interaction of G-CK with ACE-2

**Fig. 3:**
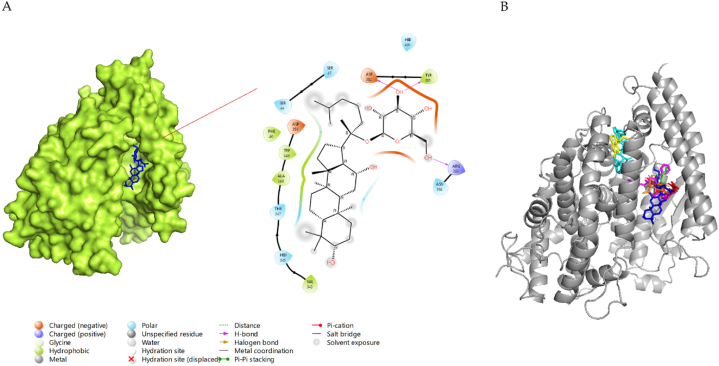
**(A)** 2D & 3D docking interactions of G-CK with ACE2 (PDB ID: 6M0J). **(B)** Docking interactions of G-CK (blue) and control drug inhibitors (Dexamethasone (Orange), NAG(Green), Remdesivir (magenta), Indinavir (cyan), Chloroquine (yellow), and Camostat (red)) with ACE2.

Angiotensin-converting enzyme 2 (ACE2), the functional receptor of SARS-CoV-2, plays a crucial role in the pathogenesis of COVID-19, as it provides viral entry into human cells [75,76]. Fig. 3-A shows the interactions between G-CK and ACE-2 with the lowest binding energy of −8.0 kcal/mol. G-CK was found to form 6-H bonds with residues ASP350, ASP382, TYR385, ASN394, ARG393, and HIS401. In addition, G-CK built other hydrophobic interactions (pi-sigma) with TRP349 residue. The binding energy of G-CK to the active site is even smaller than that of the most control molecule, indicating that G-CK has a higher binding activity. The binding site of G-CK is similar to that of the control molecule NAG, and it also shares 5-H bonds, the same as NAG (ASP350, ASP382, TYR385, ARG393, HIS401).

In addition, G-CK also shares H-bond similarities with the other control drugs such as dexamethasone (3 H-bond (ASP382, TYR385, HIS401), indinavir (2 H-Bonds (TYR385, ASP350) and camostat (5 H-Bonds (ASP350, ASP382, TYR385, ASN394, HIS401). Camostat is known to inhibit TMPRSS2 and ACE2 and protect viral fusion with the host [77]. On the other hand, the binding site and the binding pockets of G-CK were also similar to the aforementioned control drugs (Fig. 3-B). Interestingly, G-CK forms an interaction with the amino acid residue ARG393, which has been previously reported to be involved in the interaction (H-bond) with the RBD of the spike protein [59]. Notably, G-CK binds to the same or closest binding pockets predicted using the DoGSiteScorer server (Figs. S1–A). Chloroquine and remdesivir do not share similar interactions with G-CK, which shows that they might have a different mode of inhibition. Therefore, binding of G-CK at the allosteric site of the TMPRSS2 receptor may decrease biophysical interactions (e.g., electrostatic, hydrogen bonding) with the viral RBD. The representation of 2D interactions and the amino acid residues of all molecules against TMPRSS2 is shown in Fig. S2 and Table 1, and the 3D interaction of AA residues of G-CK with TMPRSS2 is shown in Fig. 5-A.

**Table 1:** Interaction of G-CK with amino acid residues of ACE2 (6M0J).

Protein	Compound	Binding Energy (kcal/mol)	H-Bond Interactions	Other Interactions	No. of H-Bond
ACE2	G-CK	−8.0	ASP350, ASP382, TYR385, ASN394, ARG393, HIS401	TRP349	6
	Dexamethasone	−7.8	SER47, ASP382, TYR385, HIS401	–	4
	Indinavir	−9.0	ALA348, ASP350, TYR385	THR347, TRP349, ASP382, ARG393, HIS401	3
	Remdesivir	−8.1	ASP206, LYS562	LEU95, TRP203, VAL209, PRO565	2
	Camostat	−7.4	SER43, ASP350, ASP382, TYR385, ASN394, HIS401	SER47, MET62, TRP349	6
	Chloroquine	−6.5	TYR196	TYR202, GLY205, GLU208, GLU564	1
	NAG	−6.2	ASP350, ASP382, TYR385, ARG393, HIS401	PHE40, ALA348	5

From the point of view of binding energy, as well as having almost similar H-bond and other types of interaction to the controls at the allosteric site of ACE2, G-CK shows strong interactions with the main host-based target ACE2 of the new coronavirus, showing G-CK might be a promising ACE2 inhibitor (Fig. 1). Hence, the G-CK and Dexamethasone in complex with ACE2 has been further utilized for molecular dynamics simulation to validate the docking scores and other properties.

#### 3.2.2 Interaction of G-CK with TMPRSS2

**Fig. 4:**
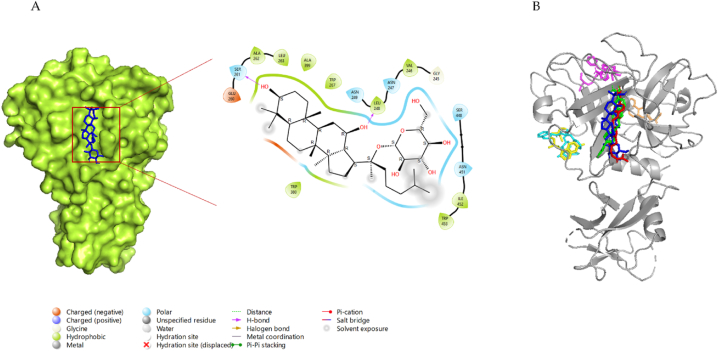
**(A)** 2D & 3D docking interactions of G-CK with TMPRSS2 (PDB ID: 7MEQ). **(B)** Docking interactions of G-CK (blue) and control drug inhibitors (Dexamethasone (Orange), Nafamostat (Green), Remdesivir (magenta), Indinavir (cyan), Chloroquine (yellow), and Camostat (red)) with TMPRSS2.

**Fig. 5:**
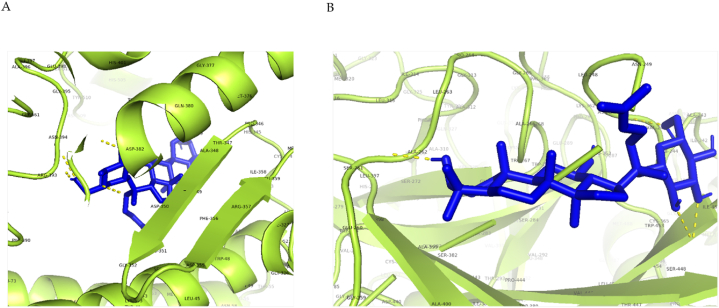
3D docking interactions of G-CK with **(A)** ACE2 **(B)** TMPRSS2.

Fig. 4-A shows the 2D and 3D interactions formed between the G-CK and TMPRSS2 with the lowest binding energy of −7.7 kcal/mol. G-CK was found to form 4-H bonds with residues LEU248, ASN247, SER261, and SER448. In addition, G-CK built other hydrophobic interactions with amino acid residue ASN249. The binding energy of the controls Dexamethasone, Indinavir, Remdesvir, Camostat, Chloroquine, and nafamostat was −6.9 kcal/mol, −7.1 kcal/mol, −7.1 kcal/mol, −7.2 kcal/mol, −5.8 kcal/mol, and −7.8 kcal/mol respectively, as shown in Table 2. The binding site of G-CK is similar to that of the control molecule nafamostat, and it shares 3-H bonds, the same as nafamostat (ASN247, SER261, SER448). Interestingly, the binding site and the binding pockets of G-CK were also similar to the aforementioned control drugs (Fig. 4-B). Notably, G-CK binds to the same or closest binding pockets predicted using DoGSiteScorer server (Figs. S1–B). The binding pockets and the binding sites were nearest to the binding pockets identified from the active site identification from the previous reports and the online tool (DoGSiteScorer). Therefore, this study reveals that G-CK can be a potential drug candidate for inhibiting TMPRSS2 and hindering the membrane fusion of the virus. The representation of 2D interactions and the amino acid residues of all molecules against TMPRSS2 is shown in Fig. S3 and Table 2, and the 3D interaction of AA residues of G-CK with ACE2 is shown in Fig. 5-B.

**Table 2:** Interaction of G-CK with amino acid residues of TMPRSS2 (7MEQ).

Protein	Compound	Binding Energy (kcal/mol)	H-Bond Interactions	Other Interactions	No. of H-Bond
TMPRSS2	G-CK	−7.7	ASN247, LEU248, SER261, SER448	ASN249	4
	Dexamethasone	−6.9	VAL298, ASN303, SER339	–	3
	Indinavir	−7.1	THR407	LEU373, GLU406, PRO422, ILE425, VAL479, MET478	1
	Remdesivir	−7.1	SER436, GLY439, SER441, GLY464	THR341, LYS342, LEU419, CYS437, GLN438, TRP461, SER463, CYS465	4
	Camostat	−7.2	ASN249, GLU260, SER261, TRP267, TRP453	GLY245, LEU263, ALA266, TRP380, SER448	5
	Chloroquine	−5.8	LEU373	ILE405, THR407, ILE425, ILE456, MET478	1
	Nafamostat	−7.8	ASN247, SER448, ASN451, GLU260, SER261	LEU263, ALA266, TRP267, TRP380, TRP453	5

### 3.3 Molecular dynamic simulation

**Fig. 6:**
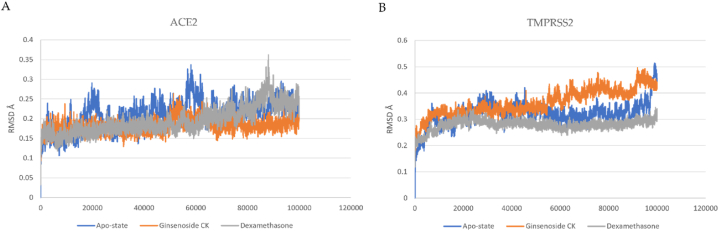
**(A)** RMSD of G-CK and Dexamethasone in complex with SARS-Cov-2 ACE2 as a function of MD simulation time. (B) RMSD of G-CK in complex with SARS-Cov-2 TMPRSS2 as a function of MD simulation time.

**Fig. 7:**
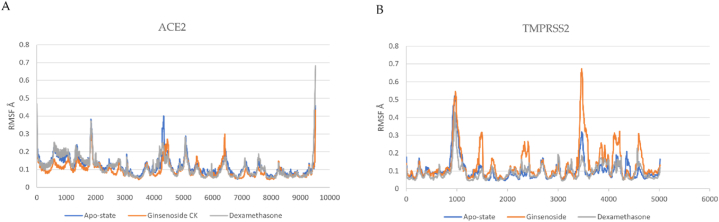
**(A)** RMSF of G-CK and Dexamethasone in complex with SARS-Cov-2 ACE2 as a function of MD simulation time. (B) RMSF of G-CK in complex with SARS-Cov-2 ACE2 as a function of MD simulation time.

**Fig. 8:**
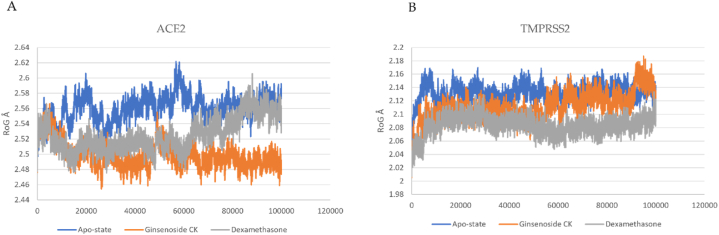
**(A)** The radius of gyration plots of molecular dynamics (MD) simulation of ACE2 receptor- G-CK and Dexamethasone complexes. (B) The radius of gyration plots of molecular dynamics (MD) simulation of ACE2 receptor G-CK and Dexamethasone complexes.

**Fig. 9:**
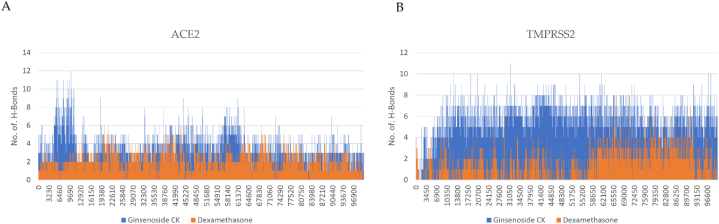
**(A)** Line plots of Ligand-protein H bonds for ACE2 with G-CK and Dexamethasone. (B) Line plots of Ligand-protein H bonds for ACE2 with G-CK and Dexamethasone.

G-CK and dexamethasone in complex with the crystal protease complex ACE-2 and TMPRSS2 have been run for 100 ns molecular dynamics simulation to investigate virtual molecular docking results further. The docked models with ACE-2 and TMPRSS2 complex with and without ligands were simulated to validate the docking scores. From that MD simulation trajectory, we have analyzed root mean square deviation (RMSD), root mean square fluctuation (RMSF), the radius of gyration (Rg), and the number of hydrogen bonds to check receptor-ligand conformational properties such as stability and flexibility. The complex of G-CK with ACE2 and TMPRSS2 had the highest docking score, and multiple amino acid residues were observed to interact with the G-CK. The protein-ligand complex was simulated for 100 ns under the NTP ensemble. RMSD and RMSF calculations were performed to analyze the trajectory generated after simulation and represented in Fig. 6, Fig. 7. The RMSD (root mean square deviation) values show that the position did not change much once the ligands had stabilized. The RMSD value of ACE2-G-CK and TMPRSS2-G-CK complex was found stable without significant deviations during the 100 ns simulation period, which was maintained below 4 Å. Both G-CK and dexamethasone showed almost same positional change and stability. The RMSF (root mean square fluctuation) values are calculated to average the fluctuations of the positions of each residue to check the flexibility and mobility of a protein during the simulation (Fig. 7 (A & B). After that, the compactness of the protein–ligand complexes analyzed by recording the ROG values were also obtained. The radius of gyration is used to study the overall effect of the ligands on the protein. The radius of gyration is calculated for each complex, as shown in Fig. 8(A and B). Further, the number of hydrogen bonds has been calculated and shown in Fig. 9 (B). MD simulation study reveals that G-CK can successfully interact with ACE-2 and TMPRSS2 to form a stable complex. This new computational approach be fast and easy for drug discovery and opened new research dimensions.

### 3.4 MM-PBSA free energy calculation

The free binding energies reveal the thermodynamic stability of docked protein–ligand complexes. It is an important property that assesses the bioactivity of ligands. The MM-PBSA analyses reveal that the average energy of the ACE2-G-CK complex (−25.89 kcal/mol) was observed to be higher than the ACE2-Dexamethasone (−13.2 kcal/mol). This indicates that G-CK is tightly bound to the receptor. The binding energy of TMPRSS2-G-CK (−7.0 kcal/mol) was less than the TMPRSS2-Dexamethasone (−13.72 kcal/mol). MM-PBSA analyses (Table 3) indicate that the ACE2-G-CK and TMPRSS2-G-CK were thermodynamically more stable than the control dexamethasone. The van der Waals energy, Δ EvdW of ACE2, was favorable to G-CK (−43.25 kcal/mol), whereas it was −23.21 kcal/mol for TMPRSS2. The MM/PBSA results suggest that ACE2-G-CK possessed the least binding energy. The ΔEEL of G-CK is similar to that of the control drug dexamethasone for ACE2. All other energies of G-CK, such as ΔEPB, ΔENPOLAR, ΔEDISPER, ΔGGAS, and ΔGSOLV for ACE2 and TMPRSS2 have shown higher binding energy than the control. Therefore, G-CK with the maximum negative binding energy and better binding affinity could be utilized as potential inhibitors against the ACE2 and TMPRSS2 of COVID-19.

**Table 3:** The calculation of binding free energy (MM-PBSA).

Complex	ACE2- G-CK Complex	ACE2- Dexamethasone Complex	TMPRSS2 – G-CK Complex	TMPRSS2 - Dexamethasone Complex
ΔVDWAALS (kcal/mol)	−43.25	−26.9	−23.21	−19.35
ΔEEL (kcal/mol)	−35.51	−35.99	−22.91	−14.14
ΔEPB (kcal/mol)	73.82	52.43	31.98	31.31
ΔENPOLAR (kcal/mol)	−30.47	−21.69	−20.21	−14.72
ΔEDISPER (kcal/mol)	61.3	45.38	42.25	30.62
ΔGGAS (kcal/mol)	−78.76	−62.91	−46.13	−33.5
ΔGSOLV (kcal/mol)	104.65	76.11	54.02	47.22
ΔTOTAL (kcal/mol)	−25.89	−13.2	−7.0	−13.72

### 3.5 ADMET properties of G-CK

**Fig. 10:**
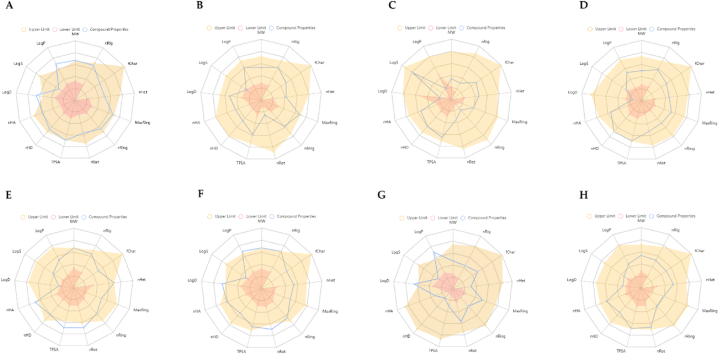
ADMET properties of G-CK and the control drugs (A) G-CK, (B) Dexamethasone, (C) NAG, (D) Nafamostat, (E) Remdesivir, (F) Indinavir, (G) Chloroquine, and (H) Camostat. *Abbreviations: MW: Molecular weight; nRig: number of rigid bonds; fChar: formal charge; nHet: number of heteroatoms; MaxRing: number of atoms in the biggest ring; nRing number of rings; nRot: number of rotatable bonds; TPSA: topological polar surface area; nHD: number of hydrogen bond donors; nHA: number of hydrogen bond acceptor; LogD: logP at physiological* pH *7.4; logS: log of the aqueous solubility; and LogP: log of the octanol/water partition coefficient.*

This study analyzed the ADME of eight drugs, including the G-CK, using the ADMETlab 2.0 [68]. This analysis represents chemical compounds' physicochemical properties and their biological functions. The resulting physicochemical and biological properties are molecular weight, number of rigid bonds, formal charge, number of heteroatoms, number of atoms, number of rotatable bonds, topological polar surface area, number of hydrogen bond donors, number of hydrogen bond acceptor, logP at physiological pH 7.4, log of the aqueous solubility, and log of the octanol/water partition coefficient (Fig. 10 (A-H). Compounds with molecular weight (MW) of ≤500 Da are preferable for oral absorption; however, compounds with higher MW are absorbed across the membrane. However, G-CK does not pass Lipinski's rule of five but Pfizer's rule. It has been revealed that natural compounds mostly do not follow Lipinski's rule [78,79], and they tend to keep their low hydrophobicity and their potential of donating the intermolecular H-bonds. To check the absorption of G-CK, the human colon adenocarcinoma cell lines (Caco-2) Permeability, Madin−Darby Canine Kidney cells (MDCK) Permeability, P-glycoprotein inhibitor (Pgp-inhibitor), Pgp-substrate, Human intestinal absorption (HIA), human oral bioavailability F20%, and F30% were computed. G-CK showed good Caco-2 and MDCK permeability. It is also an excellent Pgp inhibitor, showing good oral bioavailability (Table S5). All the other physicochemical properties of G-CK were observed to be within the acceptable limits and better than the control drugs to be considered as a lead molecule (Tables S4–S9).

### 3.6 *Invitro* cytotoxic effect of G-CK

**Fig. 11:**
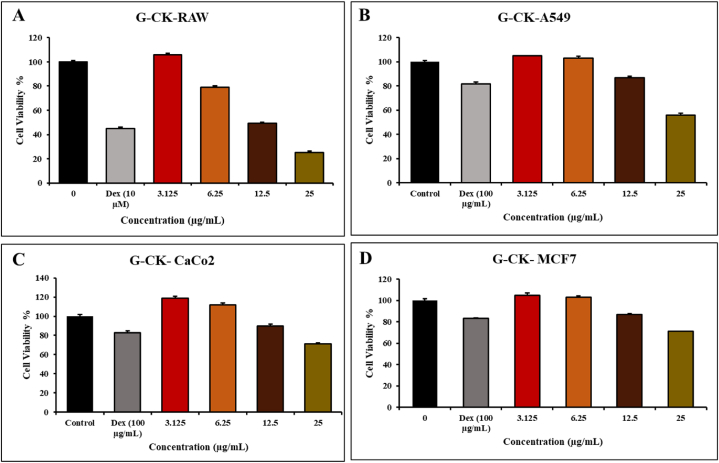
*In vitro*, cell cytotoxicity evaluation for G-CK (A) RAW 264.7, (B) A549 cells, (C) Caco-2, and (D) MCF-7 cells, cells. Every value is expressed as the mean ± standard error of three independent experiments. ***p < 0.001 compared with control.

In the previous studies, A549 cells, MCF-7 cells, and Caco-2 cells were used as a model for SARS-CoV-2 viral entry targets related to inhibition [80,81]. To investigate the inhibitory effect of G-CK on the TMPSS2 and ACE2, we used three different cancerous cells and one normal RAW 264.7 cell as the model in our study. These cell lines have been widely used in ACE2 and TMPRSS2-related research and can provide valuable insights into the relationship between ACE2, TMPRSS2, COVID-19, and cancer [82]. The cytotoxicity level of G-CK on all the cell lines such as RAW 264.7, A549 cells, MCF-7 cells, and Caco-2 cells, was studied for 24 h using various concentrations (0,3.125,6.25,12.5,25) μg/mL and dexmathanose was used as a positive control with the concentration of 100 μg/mL. In a cytotoxicity experiment, we utilized an MTT solution to measure the cell's toxicity level. After treatment at a concentration range of (1–12.5) μg/mL, G-CK did not inhibit the proliferation of A549 cells, MCF-7 cells, and Caco-2 cells at 24 h. It showed minimal cytotoxic effects on all cancerous cells at 12.5 μg/mL compared with control. Hence, the result is suggesting that this concentration range of G-CK has considerable cytotoxic effect on A549 cells, MCF-7 cells, and Caco-2 cells growth (Fig. 11 (A-D)). On the other hand, at the concentration of 6.25 μg/mL, normal cells (RAW 264.7) showed less toxicity with the sample of G-CK compared with control.

### 3.7 Effect of G-CK on ACE2 and TMPRSS2 expression in different cancer cell lines

**Fig. 12:**
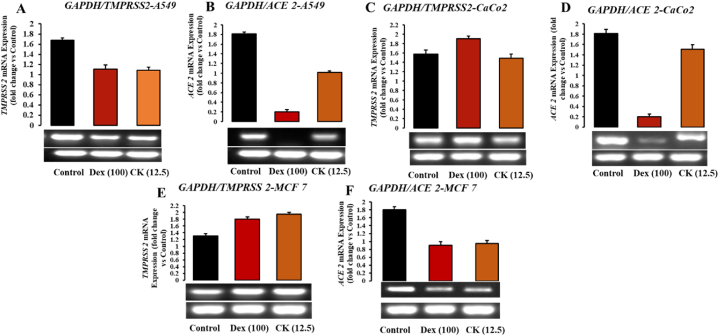
Effects of G-CK on mRNA expression levels of Anti-covid related genes in (A–B) A549 cells, (C–D) Caco-2 cells, and (E–F) MCF-7 cells.

ACE2 was discovered as a receptor for SARS-CoV infection and transmission in 2003 [83]. ACE2 expression and distribution in various organs and tissues of the human body could suggest SARS-CoV-2 infection routes. Research has indicated high ACE2 expression in the lungs, kidneys, esophagus, colon, heart, brain, and other organs [[84], [85], [86]]. The androgen-responsive serine protease TMPRSS2 is found in many tissues and organs, including the lungs, skin, and urogenital tract [87,88]. Organs with abundant ACE2 and TMPRSS2-expressing cells are considered potential sites of SARS-CoV-2 infection. Recent studies have shown significant ACE2 expression in the oral cavity, implying that the oral route may play a significant role in SARS-CoV-2 infection [89]. SARS-CoV infection disrupts the physiological equilibrium between ACE/ACE2 and the angiotensin II/angiotensin system, contributing to SARS-CoV-induced lung damage. In our present study, we investigated the effect of G-CK on ACE2 and TMPRSS2 expression in host cells, as the SARS-CoV-2 spike protein can directly bind to ACE2. RAW 264.7, A549, MCF-7, and Caco-2 cells were used as models to investigate the effect of G-CK on ACE2 and TMPRSS2 mRNA expression in host cells. Our results demonstrated that A549, MCF-7, and CaCo-2 cells express ACE2. Treating the cells with G-CK at a concentration of 12.5 μg/mL (Fig. 12 (A-F)) modulated the expression of both enzymes. Specifically, ACE2 was reduced significantly in A549 and MCF-7 cells, similar to the control drug dexamethasone. CaCo-2 cells showed a slight reduction in ACE2 mRNA levels, while dexamethasone exhibited a higher level of inhibition. Regarding TMPRSS2, A549, and CaCo-2 cells, the expression levels were higher, while MCF-7 cells showed slightly lower mRNA levels, as shown in Fig. S1. This differential expression of TMPRSS2 in MCF-7 is consistent with the results of Xiao et al. [90]. It has been reported that higher levels of TMPRSS2 in MCF-7 could potentially affect breast cancer prognosis, whereas reduced expression of TMPRSS2 in A549 may affect lung cancer prognosis. Therefore, in addition to the inhibitory potential of G-CK on SARS-CoV-2 targets, our study reveals a potential role of G-CK in modulating TMPRSS2 expression in lung and breast cancer, opening up a new research direction. However, the exact cause of this differential expression of TMPRSS2 in different tissues is not fully understood and requires further investigation.

G-CK significantly inhibited the mRNA level of TMPRSS2 in A549 cells, but the expression was increased in MCF-7 cells, while no changes were observed in CaCo-2 cells. Finally, these results suggest that 12.5 μg/mL of G-CK could significantly inhibit the mRNA expression of ACE2 in A549 and MCF-7 cells. Similarly, the level of TMPRSS2 was reduced in A549 but increased in MCF-7 and CaCo-2 cells at the same concentration, suggesting the involvement of alternative pathways and genes (i.e., steroid receptors). Remarkably, as discussed earlier, G-CK exhibited a similar pattern of mRNA levels to the control drug dexamethasone, confirming G-CK as a natural glucocorticoid that could potentially serve as a replacement for dexamethasone in treating various diseases.

### 3.8 Effect of G-CK on ACE2 and TMPRSS2 expression in LPS-induced RAW 264.7 cells

To observe and validate the inhibitory potential of G-CK in non-cancerous cells and investigate the relationship between ACE2, TMPRSS2 overexpression, and cytokine storm, we induced the overexpression of ACE2 and TMPRSS2 in RAW 264.7 cells using LPS (Lipopolysaccharide). LPS induces an increase in intracellular ROS production, which subsequently enhances AKT phosphorylation and leads to increased expression of HIF-1α (Hypoxia-inducible factor 1-alpha). This, in turn, upregulates ACE2 expression. Interestingly, the levels of both ACE2 and TMPRSS2 increased following treatment with LPS, suggesting a possible involvement of ACE2 and TMPRSS2 in immunomodulation in COVID-19 and cancer.

**Fig. 13:**
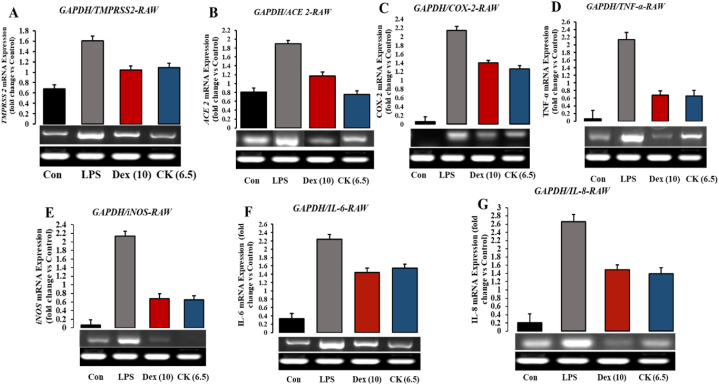
Effects of G-CK on mRNA expression levels of Anti-covid (A–B) and Cytokine storm (C–G) related genes on Raw 264.7 cells compared with LPS.

Upon treatment with G-CK, the mRNA levels of both ACE2 and TMPRSS2 were significantly reduced at a concentration of 6.25 μg/mL. In RAW 264.7 cells, G-CK effectively inhibited the mRNA expression of ACE2 and TMPRSS2, similar to the control drug (Fig. 13 (A-B)). Notably, the reduction in ACE2 mRNA expression was higher compared to dexamethasone, shown in Fig. S2(A-B). Therefore, it is apparent that the levels of ACE2 and TMPRSS2 and the action of G-CK in RAW 264.7 cells, differ from those in cancer cells.

### 3.9 Effect of G-CK on cytokine expression in LPS-induced RAW 264.7 cells

To assess the inhibitory potential of G-CK on the overexpression of cytokines associated with cytokine storm in COVID-19, we induced RAW 264.7 cells with LPS. Cytokines, including COX-2, TNF-α, iNOS, IL-6, and IL-8, were evaluated. The results demonstrated that consistent with previous studies, G-CK significantly inhibited most cytokines, similar to dexamethasone. Moreover, at a concentration of 6.25 μg/mL (Fig. 13 (C-G)), the mRNA expression of COX-2, TNF-α, iNOS, IL-6, and IL-8 was notably downregulated in the LPS-treated group shown in Fig. S2(C–F). Notably, TNF-α, known to contribute to cytokine storms crucially, was significantly suppressed. In conclusion, G-CK has demonstrated potential in treating cytokine storms in COVID-19 inhibiting the host-based viral entry targets and suppressing the overproduction of cytokines, thus preventing cytokine storms and reducing the severity of the disease.

## 4 Conclusion

The inhibition of ACE2 and TMPRSS2 protein represents a promising strategy for controlling viral entry and membrane fusion, leading to the discovery of potential drug candidates. The molecular docking and dynamics analysis with G-CK showed highly favorable interactions on ACE2 and TMPRSS2 involving ligand-protein interaction and good docking scores with higher complex stability. From the comparative analysis, G-CK showed better results than the standard ACE2 and TMPRSS2 inhibitors. Moreover, the proposed potential inhibitors also meet the criteria of drug-likeness based on the ADME properties. *In vitro* results from our study showed that G-CK significantly decreased ACE2 expression in a dose-dependent manner in A549 and RAW 264.7 cells. When G-CK was administered, the mRNA levels of both ACE2 and TMPRSS2 were notably decreased. In RAW 264.7 cells, G-CK exhibited effective inhibition of ACE2 and TMPRSS2 mRNA expression, similar to the control drug. It is worth mentioning that the reduction in ACE2 mRNA expression was more remarkable compared to dexamethasone. This suggests that the levels of ACE2 and TMPRSS2 and the impact of G-CK in RAW 264.7 cells, differ from those observed in cancer cells. In A549 cells, G-CK demonstrated significant inhibition of TMPRSS2 mRNA levels, whereas an increase in expression was observed in MCF-7 cells. No noticeable changes were detected in CaCo-2 cells. Remarkably, the findings indicate that a 12.5 μg/mL concentration of G-CK effectively reduced the mRNA expression of ACE2 in both A549 and MCF-7 cells. Similarly, at the same concentration, the level of TMPRSS2 was decreased in A549 cells but increased in MCF-7 and CaCo-2 cells, suggesting the involvement of alternative pathways and genes, such as steroid receptors. It is worth noting that, as mentioned earlier, G-CK displayed a mRNA expression pattern similar to the control drug dexamethasone, underscoring G-CK as a natural glucocorticoid with potential to be used as a substitute for dexamethasone in the treatment of various diseases. Moreover, at a concentration of 6.25 μg/mL (Fig. 13), the mRNA expression of COX-2, TNF-α, iNOS, IL-6, and IL-8 was notably downregulated in the LPS-treated group.

In conclusion, our results suggest that G-CK could be a candidate for prophylaxis and treatment of SARS-CoV-2 infection by inhibiting ACE2 and TMPRSS2 expression *in silico* and *in vitro*. However, the exact clinical effect remains unknown. Hence, further experimental and clinical investigations are required to reveal G-CK's complete inhibitory potential. In addition, all available ginsenosides from *Panax Ginseng* should be explored for their SARS-CoV-2 anti-viral efficacy in the future.

## Author contributions

V.B and S.C.K: Conceptualization; V.B and J.N: Data curation; V.B, M.M, and B.M.K: Formal analysis; D.C.Y, S.C.K, and R.M: Funding acquisition; S.K.C, C.S.L, and D.U.Y: Investigation; V.B and J.N: Methodology; S.C.K and R.M: Project administration; D.C.Y: Resources and Software; V.B and J.N: Roles/Writing - original draft; V.B: Writing - review & editing.

## Funding and acknowledgments

This work was supported by Korea Institute of Planning and Evaluation for Technology in Food, Agriculture and Forestry (IPET) through Agri-Food Export Business Model Development Program, funded by Ministry of Agriculture, Food and Rural Affairs (MAFRA) (Project No: 320104-03). This research was funded by the Science and technology development project of Jilin Province (NO. 20210509022RQ). This research was supported by the Basic Science Research Program through the National Research Foundation of Korea (NRF) funded by the Ministry of Education (grant no: NRF- 2020R1I1A1A01070867).

## Data availability

Data is presented within the manuscript and the supplemental materials.

## Declaration of competing interest

The authors declare that they have no known competing financial interests or personal relationships that could have appeared to influence the work reported in this paper.
