# Supplementary material for: In silico and in vitro inhibition of host-based viral entry targets and cytokine storm in COVID-19 by ginsenoside compound K
Source: Heliyon. 2023 Aug 20;9(9):e19341. doi: 10.1016/j.heliyon.2023.e19341 (PMC10558348; doi:10.1016/j.heliyon.2023.e19341)
Supplement: Multimedia component 1 [file mmc1.docx]

*Research article*

***In Silico* and *In Vitro* Inhibition of Host-Based Viral Entry Targets and Cytokine Storm in COVID-19 by Ginsenoside Compound K**

Vinothini Boopathi^1^†, Jinnatun Nahar^1^†, Mohanapriya Murugesan^1^, Sathiyamoorthy Subramaniyam^4^, Byoung Man Kong^2^, Sung-Keun Choi^3^, Chang-Soon Lee^3^, Li Ling^2^, Dong Uk Yang^1^, Deok Chun Yang^1,2^ , Ramya Mathiyalagan^1 *^ and Se Chan Kang^1,2, *^

^1^ Graduate School of Biotechnology, College of Life Sciences, Kyung Hee University, Yongin-si, Gyeonggi-do 17104, Korea; vinothini9327@gmail.com (V.B.); jinnatunnaharbph@gmail.com (J.N); priyabuddy44@gmail.com (M.M.); rudckfeo23@naver.com (D.U.Y.); dcyang@khu.ac.kr (D.C.Y.); sckang@khu.ac.kr (S.C.K.); ramyabinfo@gmail.com (R.M.).

^2^ Department of Oriental Medicinal Biotechnology, College of Life Science, Kyung Hee University, Yongin-si, Gyeonggi-do 17104, Korea; kong2167@naver.com (B.M.K); aqling@naver.com (L.L).

^3^ Daedong Korea Ginseng Co., Ltd, 86, Gunbuk-ro, Gunbuk-myeon, Geumsan-gun, Chungcheongnam-do 32718 Republic of Korea; ddgc0815@ddkorea.co.kr (S.K.C); hippo8270@ddkorea.co.kr (C.S.L).

^4^ Research and Development Center, Insilicogen Inc., Yongin, Republic of Korea; s.sathiyamurthi@gmail.com (S.S.).

*Correspondence: sckang@khu.ac.kr (S.C.K.) and ramyabinfo@gmail.com (R.M.)

† Equally contributed.


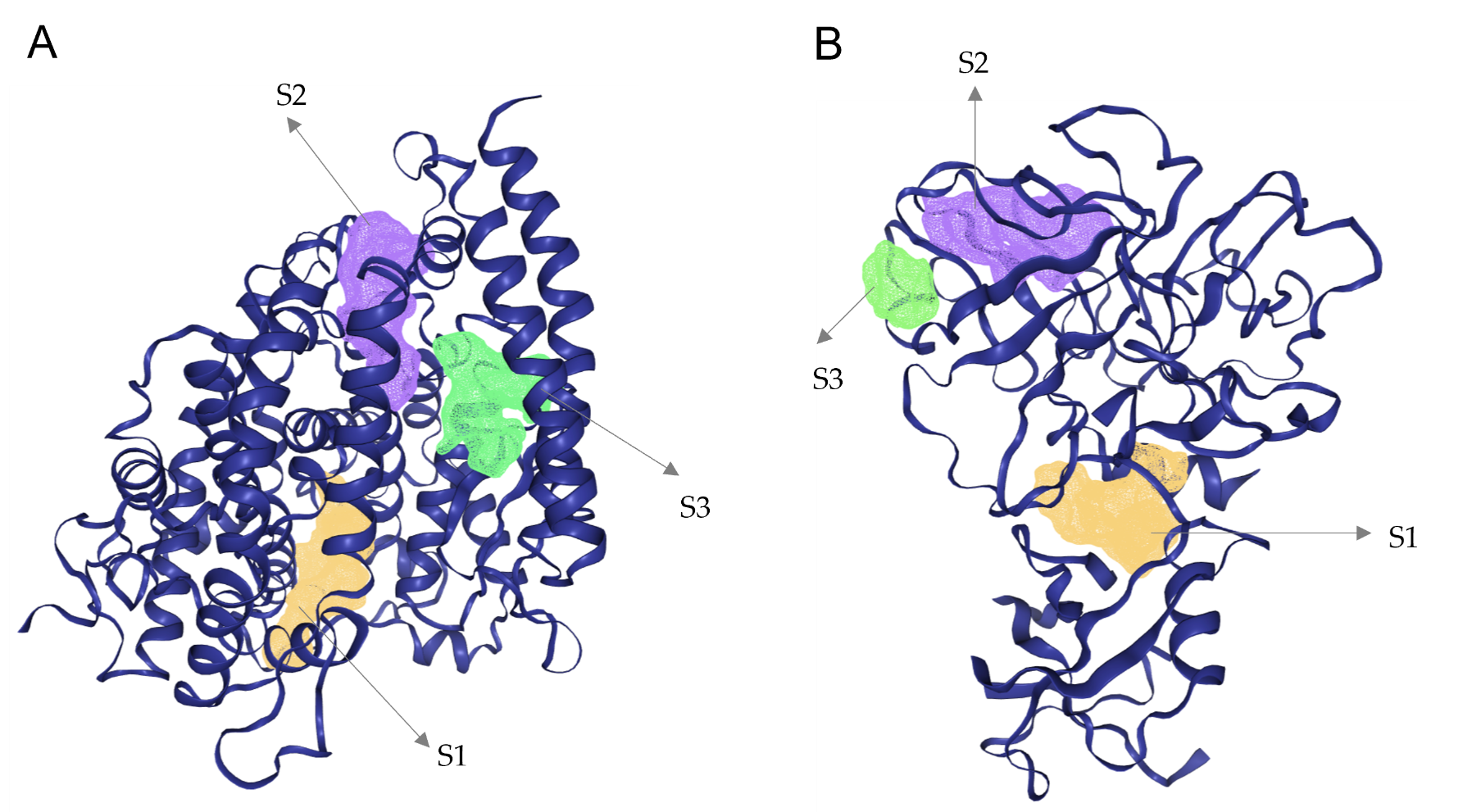


**Figure S1**. Predicted active site for (A) ACE2 and (B) TMPRSS2 (DoGSiteScorer webserver)


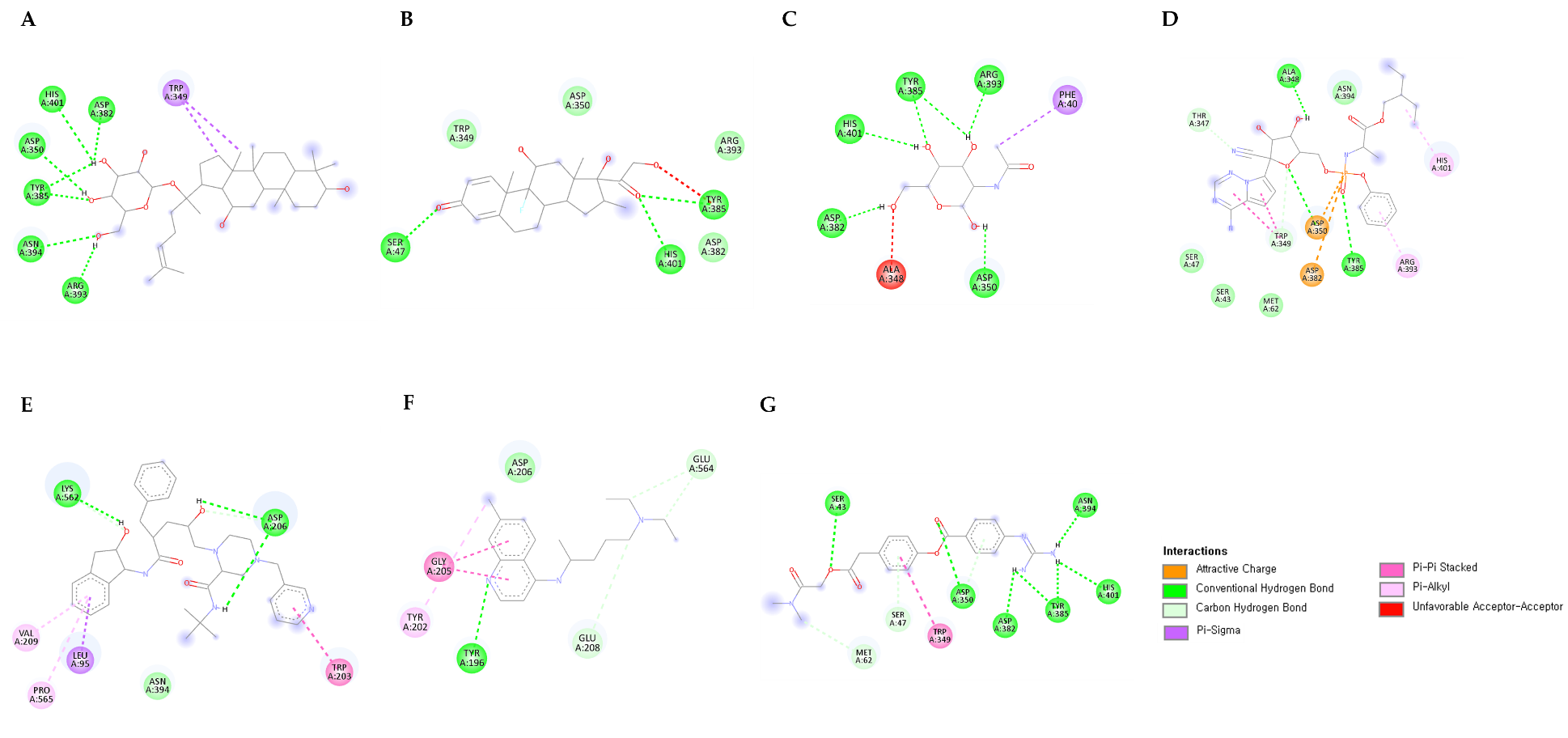


**Figure S2.** 2D interaction diagram of ACE2 with (A) Ginsenoside CK, (B) Dexamethasone, (C) NAG, (D) Remdesivir, (E) Indinavir, (F) Chloroquine, and (G) Camostat.

**
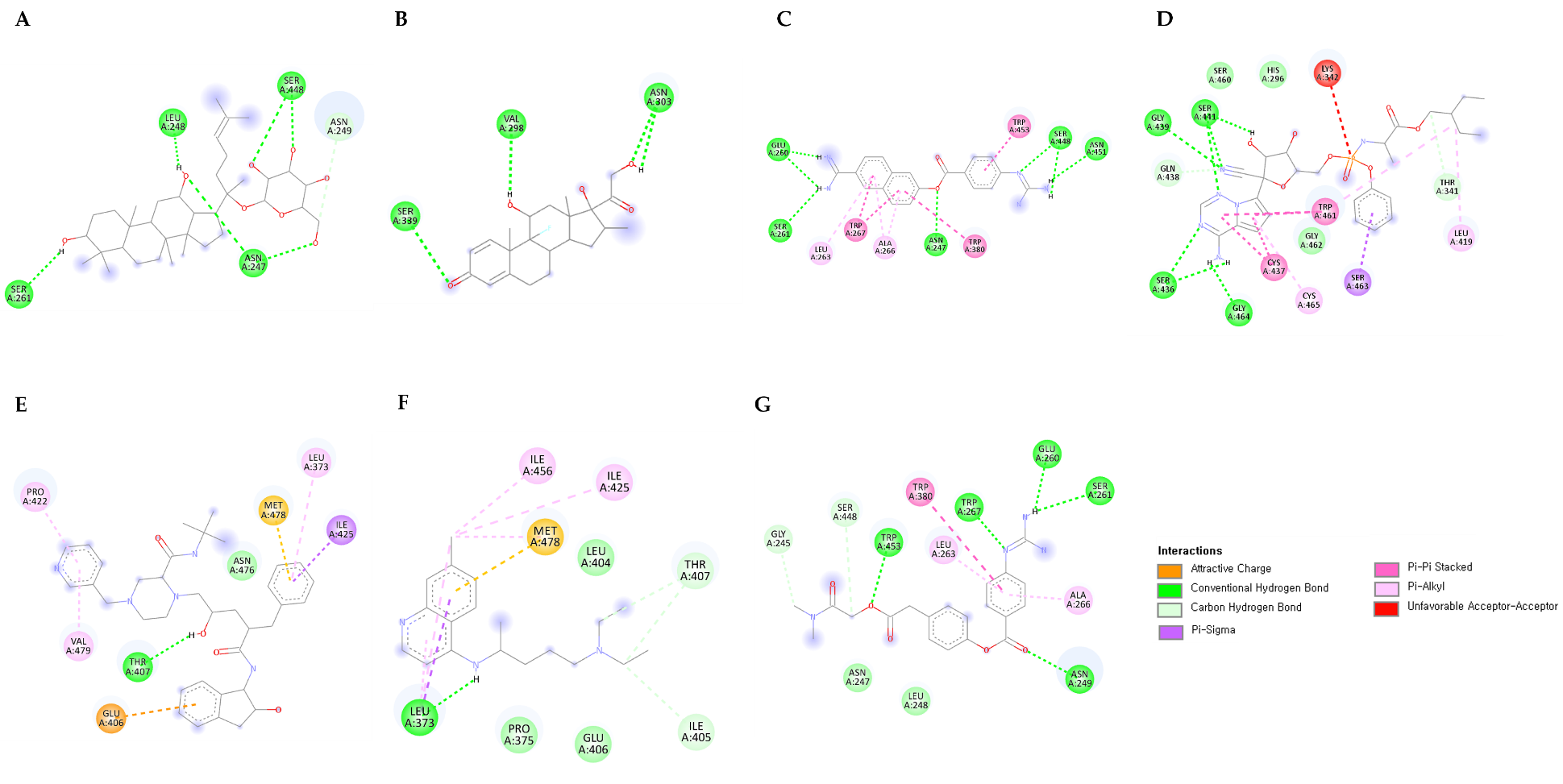
**

**Figure S3.** 2D interaction diagram of TMPRSS2 with (A) Ginsenoside CK, (B) Dexamethasone, (C) Nafamostat, (D) Remdesivir, (E) Indinavir, (F) Chloroquine, and (G) Camostat


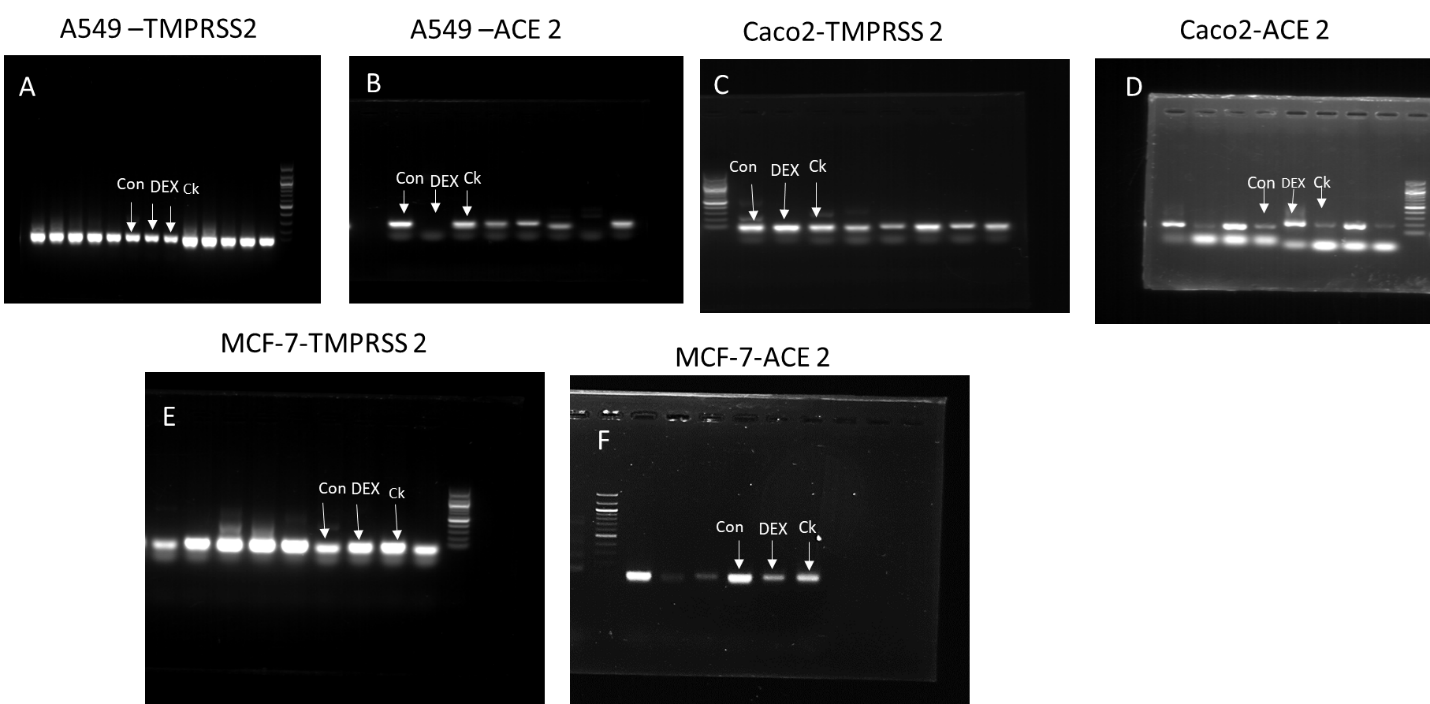


**Figure S4:** Anti-covid related genes Expression in (A-B) A549 cells, (C-D) Caco-2 cells and (E-F) MCF-7 cells.


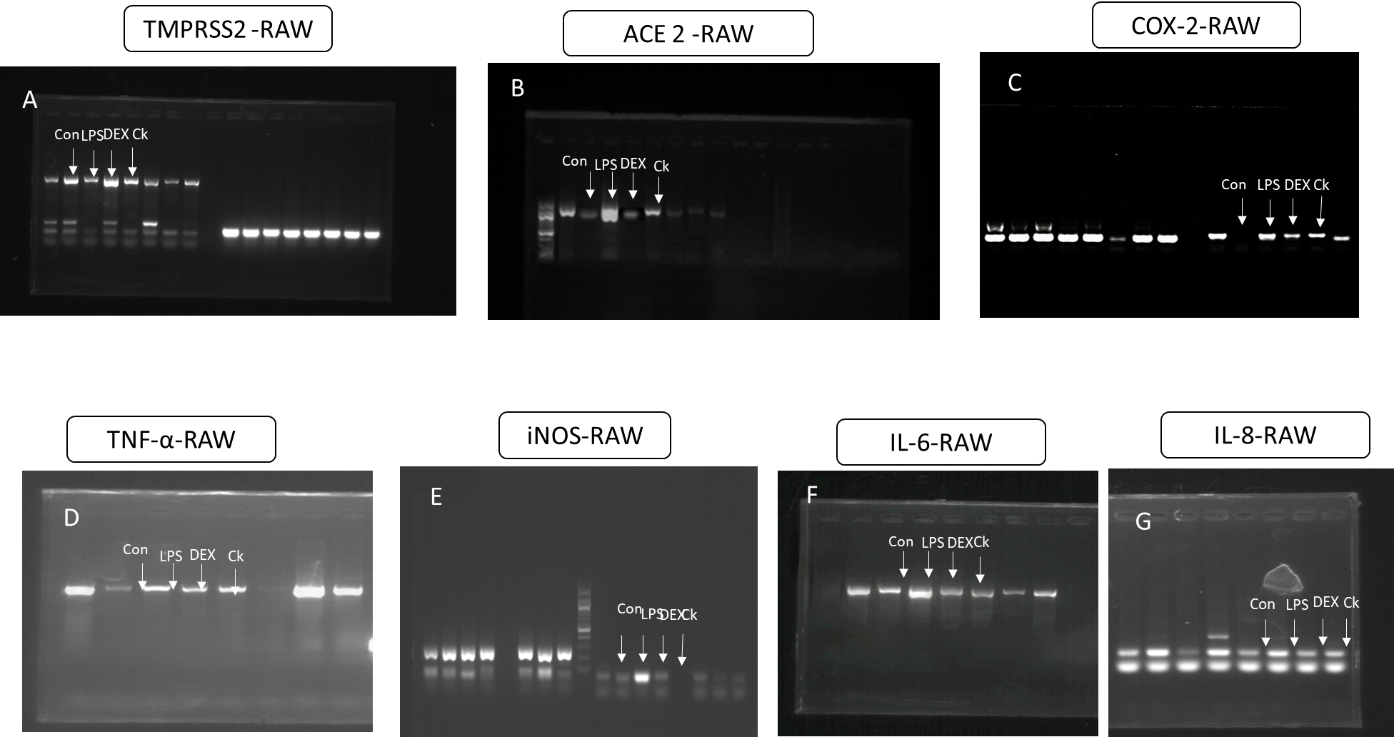


**Figure S5**: Effects of G-CK on mRNA expression levels of Anti-covid (A-B) and Cytokine storm (C-G) related genes on Raw 264.7 cells compare with LPS.

**Table S1. Grid box coordinates and size parameters used for molecular docking.**

|  | ACE2 | TMPRSS2 |  |
| --- | --- | --- | --- |
| Dimension (Å) | | | |
| *x* | 90 | 66 |  |
| *y* | 124 | 92 |  |
| *z* | 94 | 72 |  |
|  | | | |
| Centre (Å) | | | |
| *x* | −21.85 | −1.726 |  |
| *y* | 20.018 | -7.316 |  |
| *z* | -14.44 | 16.226 |  |

**Table S2. Active site prediction for ACE2 and TMPRSS2 from the literature.**

| Protein | Interacting Amino Acid Residues |
| --- | --- |
| ACE2 | Lys31, Gln24, Thr27, Phe28, Lys31, Arg357, Glu37, Gln325, Glu329, Asn330, Lys 353, Arg393, Leu29, Asp38, Tyr41, Gln42, Leu45, Lys3, His34, Gln24, Tyr83, Leu79, Met82, Glu35, His34, Gly354, Asp355, and Asp30 |
| TMPRSS2 | Arg255, Ile256, His 296, Asp 345, Ser 441, Asp435, Ser460, Gly462, Glu299, Lys300, Tyr337, Lys340, Thr341, Lys342, Asn343, Glu389, Asn418, Leu419, Ile420, Met424,Ser436,Cys437,Gln438,Trp461,Ser463,Gly464,Cys465,Val473, Asp440 |

**Table S3. Active site prediction for ACE2 and TMPRSS2 using DoGSiteScorer.**

| Protein | Site | Volume Å^3^ | Surface Å^2^ | Drug Score | Simple Score |
| --- | --- | --- | --- | --- | --- |
| ACE2 | S1 | 876.74 | 1068.47 | 0.83 | 0.56 |
|  | S2 | 639.94 | 896.33 | 0.73 | 0.42 |
|  | S3 | 484.94 | 570.92 | 0.74 | 0.0 |
|  |  |  |  |  |  |
| TMPRSS2 | S1 | 550.78 | 671.23 | 0.82 | 0.29 |
|  |  |  |  |  |  |
|  | S2 | 369.02 | 472.67 | 0.73 | 0.14 |
|  |  |  |  |  |  |
|  | S3 | 128.51 | 240.93 | 0.23 | 0.0 |
|  |  |  |  |  |  |

**Table S4. Parameters evaluated for drug-likeness of Compound K and the control drugs.**

| **Compound** | **MCE-18** | **SAscore** | **Fsp^3^** | **PAINS** | **NPScore** | **Pfizer** |
| --- | --- | --- | --- | --- | --- | --- |
| **Compound K** | 110.400 | 5.404 | 0.944 | 0 | 2.838 | Accepted |
| **Dexamethasone** | 81.579 | 4.877 | 0.727 | 0 | 2.225 | Accepted |
| **NAG** | 24.0 | 3.628 | 0.875 | 0 | 2.019 | Accepted |
| **Nafamostat** | 18.0 | 2.217 | 0.0 | 0 | -0.389 | Accepted |
| **Remdesivir** | 92.8 | 5.111 | 0.481 | 0 | 0.217 | Accepted |
| **Indinavir** | 101.189 | 4.09 | 0.472 | 0 | -0.416 | Accepted |
| **Chloroquine** | 24.0 | 3.369 | 0.5 | 0 | -0.891 | Rejected |
| **Camostat** | 15.0 | 2.28 | 0.2 | 0 | -0.844 | Accepted |
|  |  |  |  |  |  |  |

**Table S5. Parameters evaluated for absorption of Compound K and the control drugs.**

| **Compound** | **Caco-2 permeability** | **MDCK permeability** | **Pgp-inhibitor** | **Pgp-substrate** | **HIA** | **F (20%)** | **F (30%)** |
| --- | --- | --- | --- | --- | --- | --- | --- |
| **Compound K** | -4.944 | 3.1e-05 | 0.802 | 0.01 | 0.816 | 0.178 | 0.712 |
| **Dexamethasone** | -4.77 | 2.1e-05 | 0.006 | 0.005 | 0.042 | 0.012 | 0.004 |
| **NAG** | -5.386 | 0.001247 | 0.001 | 0.64 | 0.911 | 0.079 | 0.723 |
| **Nafamostat** | -6.105 | 8e-06 | 0.027 | 0.999 | 0.888 | 0.952 | 1.0 |
| **Remdesivir** | -5.996 | 4e-06 | 0.022 | 0.938 | 0.91 | 0.105 | 0.78 |
| **Indinavir** | -5.392 | 2.1e-05 | 0.978 | 0.264 | 1.0 | 0.729 | 0.089 |
| **Chloroquine** | -4.545 | 1.1e-05 | 0.752 | 0.985 | 0.002 | 0.069 | 0.013 |
| **Camostat** | -5.391 | 2.7e-05 | 0.755 | 0.9 | 0.008 | 0.712 | 0.337 |

**Table S6. Parameters evaluated for distribution of Compound K and the control drugs.**

| **Compound** | **PPB (%)** | **VD (L/kg)** | **BBB (log BB)** | **Fu (%)** |
| --- | --- | --- | --- | --- |
| **Compound K** | 93.57 | 1.061 | 0.058 | 5.597 |
| **Dexamethasone** | 62.92 | 0.81 | 0.996 | 35.91 |
| **NAG** | 9.992 | 0.348 | 0.387 | 88.28 |
| **Nafamostat** | 88.60 | 2.087 | 0.476 | 18.60 |
| **Remdesivir** | 46.71 | 1.71 | 0.245 | 43.18 |
| **Indinavir** | 91.29 | 1.032 | 0.615 | 7.303 |
| **Chloroquine** | 71.16 | 3.738 | 0.914 | 25.79 |
| **Camostat** | 67.96 | 0.684 | 0.792 | 47.83 |

**Table S7. Parameters evaluated for metabolism of Compound K and the control drugs.**

| **Compound** | **CYP1A2 inhibitor** | **CYP1A2 substrate** | **CYP2C19 inhibitor** | **CYP2C19 substrate** | **CYP2C9 inhibitor** | **CYP2C9 substrate** | **CYP2D6 inhibitor** | **CYP2D6 substrate** | **CYP3A4 inhibitor** | **CYP3A4 substrate** |
| --- | --- | --- | --- | --- | --- | --- | --- | --- | --- | --- |
| **Compound K** | 0.001 | 0.13 | 0.002 | 0.845 | 0.009 | 0.229 | 0.0 | 0.141 | 0.032 | 0.099 |
| **Dexamethasone** | 0.005 | 0.479 | 0.02 | 0.777 | 0.015 | 0.134 | 0.002 | 0.055 | 0.708 | 0.905 |
| **NAG** | 0.004 | 0.048 | 0.016 | 0.098 | 0.001 | 0.186 | 0.003 | 0.107 | 0.006 | 0.018 |
| **Nafamostat** | 0.164 | 0.139 | 0.034 | 0.059 | 0.037 | 0.027 | 0.028 | 0.33 | 0.021 | 0.079 |
| **Remdesivir** | 0.021 | 0.204 | 0.38 | 0.073 | 0.306 | 0.899 | 0.831 | 0.135 | 0.493 | 0.603 |
| **Indinavir** | 0.02 | 0.07 | 0.4 | 0.91 | 0.309 | 0.795 | 0.529 | 0.792 | 0.952 | 0.93 |
| **Chloroquine** | 0.623 | 0.967 | 0.048 | 0.356 | 0.002 | 0.065 | 0.977 | 0.921 | 0.01 | 0.45 |
| **Camostat** | 0.281 | 0.168 | 0.051 | 0.08 | 0.039 | 0.067 | 0.069 | 0.609 | 0.056 | 0.223 |

**Table S8. Parameters evaluated for excretion of Compound K and the control drugs.**

| **Compound** | **CL** | **T_1/2_** |
| --- | --- | --- |
| **Compound K** | 4.798 | 0.029 |
| **Dexamethasone** | 3.884 | - |
| **NAG** | 1.676 | 0.804 |
| **Nafamostat** | 1.45 | 0.657 |
| **Remdesivir** | 3.434 | 0.871 |
| **Indinavir** | 7.186 | 0.525 |
| **Chloroquine** | 5.831 | 0.07 |
| **Camostat** | 7.163 | 0.467 |

**Table S9. Parameters evaluated for toxicity of Compound K and the control drugs.**

| **Compound** | **hERG Blockers** | **H-HT** | **DILI** | **AMES Toxicity** | **ROA** | **Skin Sensitization** | **Carcinogenicity** | **Eye Corrosion** | **Respiratory Toxicity** |
| --- | --- | --- | --- | --- | --- | --- | --- | --- | --- |
| **Compound K** | 0.046 | 0.27 | 0.006 | 0.041 | 0.173 | 0.064 | 0.007 | 0.003 | 0.929 |
| **Dexamethasone** | 0.026 | 0.273 | 0.094 | 0.066 | 0.447 | 0.036 | 0.848 | 0.003 | 0.979 |
| **NAG** | 0.012 | 0.338 | 0.299 | 0.075 | 0.011 | 0.04 | 0.008 | 0.003 | 0.011 |
| **Nafamostat** | 0.538 | 0.598 | 0.8 | 0.058 | 0.733 | 0.376 | 0.662 | 0.003 | 0.691 |
| **Remdesivir** | 0.65 | 0.781 | 0.888 | 0.779 | 0.799 | 0.188 | 0.096 | 0.003 | 0.968 |
| **Indinavir** | 0.511 | 0.841 | 0.041 | 0.015 | 0.518 | 0.035 | 0.033 | 0.003 | 0.563 |
| **Chloroquine** | 0.953 | 0.766 | 0.394 | 0.394 | 0.201 | 0.949 | 0.039 | 0.004 | 0.986 |
| **Camostat** | 0.334 | 0.175 | 0.942 | 0.116 | 0.162 | 0.317 | 0.328 | 0.003 | 0.009 |
